# Vector Interactions and Molecular Adaptations of Lyme Disease and Relapsing Fever Spirochetes Associated with Transmission by Ticks

**DOI:** 10.3201/eid0802.010198

**Published:** 2002-02

**Authors:** Tom G. Schwan, Joseph Piesman

**Affiliations:** * National Institutes of Health, Hamilton, Montana, USA; † Centers for Disease Control and Prevention, Fort Collins, Colorado, USA

**Keywords:** Lyme disease, relapsing fever, ticks, surface proteins, Borrelia

## Abstract

Pathogenic spirochetes in the genus *Borrelia* are transmitted primarily by two families of ticks. The Lyme disease spirochete, *Borrelia burgdorferi*, is transmitted by the slow-feeding ixodid tick *Ixodes scapularis,* whereas the relapsing fever spirochete, *B. hermsii*, is transmitted by *Ornithodoros hermsi*, a fast-feeding argasid tick. Lyme disease spirochetes are generally restricted to the midgut in unfed *I. scapularis*. When nymphal ticks feed, the bacteria pass through the hemocoel to the salivary glands and are transmitted to a new host in the saliva after 2 days. Relapsing fever spirochetes infect the midgut in unfed *O. hermsi* but persist in other sites including the salivary glands. Thus, relapsing fever spirochetes are efficiently transmitted in saliva by these fast-feeding ticks within minutes of their attachment to a mammalian host. We describe how *B. burgdorferi* and *B. hermsii* change their outer surface during their alternating infections in ticks and mammals, which in turn suggests biological functions for a few surface-exposed lipoproteins.

The molecular adaptations required by pathogenic spirochetes for efficient transmission by obligate, blood-feeding ticks are largely unknown. In the new era of genomics, the complete DNA sequence of two spirochetes, *Borrelia burgdorferi* and *Treponema pallidum*, have been determined (*1*,*2*). As additional genome sequences become available for other pathogenic and free-living spirochetes, comparisons of their genomes may elucidate genes that are unique to those species of spirochetes associated with ticks. This information, along with an increased understanding of the molecular mechanisms used by tick-borne spirochetes to adapt for transmission by their tick vectors, may lead to unique disease prevention strategies.

The genus *Borrelia* currently contains 37 species of spirochetes, many of which cause diseases in humans and domestic animals (Table) (*3*). Except for *Borrelia recurrentis* (which causes louse-borne relapsing fever and is transmitted by the human body louse), all known species are transmitted by ticks (*4*). Two groups of spirochetes stand out among these tick-borne species because of their prevalence as human pathogens: Lyme disease spirochetes, transmitted by the relatively slow-feeding ixodid (hard) ticks of the genus *Ixodes*, and relapsing fever spirochetes, transmitted by the fast-feeding argasid (soft) ticks of the genus *Ornithodoros* (Figure 1). Major observations in recent years have increased our understanding of how one species in each group adapts while infecting ticks. *B. burgdorferi*, a causative agent of Lyme disease, and *B. hermsii*, a causative agent of tick-borne relapsing fever, have received the most attention. We describe how these two species of *Borrelia* change their outer surface during their alternating infections in ticks and mammals, which in turn suggests biological functions for a few surface-exposed lipoproteins. The dynamics of infection of these two bacteria in strikingly different types of ticks provide examples of possible adaptations for their transmission.

**Table:** Diseases caused by infection with *Borrelia* species

Disease	No. of species	Arthropod vector
Lyme disease	3^a^	Hard ticks (*Ixodes* spp.)
Tick-borne relapsing fever	21^b^	Soft ticks (*Ornithodoros* spp.)
Avian borreliosis	1^c^	Soft ticks (*Argas* spp.)
Bovine borreliosis	1^d^	Hard ticks (*Boophilus* spp.)
Louse-borne relapsing fever	1	Body louse (*Pediculus*)

^a^*Borrelia burgdorferi, B. garinii,* and *B. afzelii* are known human pathogens. ^b^Some species of *Borrelia* associated with *Ornithodoros* ticks are of unknown pathogenicity. ^c^*B. anserina.* ^d^*B. theileri*. ^e^*B. recurrentis.*

**Figure 1:**
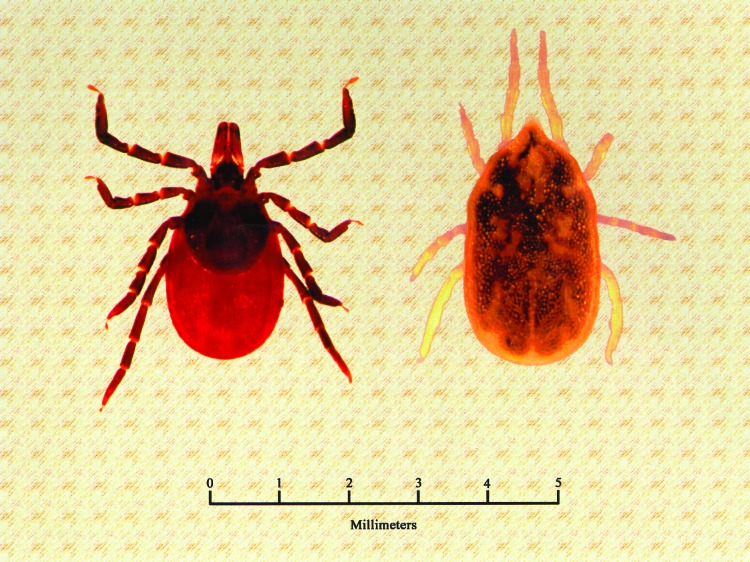
Dorsal view of a female *Ixodes scapularis* (family Ixodidae, hard ticks), a vector of *Borrelia burgdorferi* (left), and a female *Ornithodoros hermsi* (family Argasidae, soft ticks), the vector of *B. hermsii* (right).

## *B. burgdorferi*-Tick Interactions

Detailed studies of *B. burgdorferi* were initiated in 1982 when Burgdorfer and coworkers reported these bacteria in adult *Ixodes scapularis* ticks collected from vegetation on Shelter Island, New York (*5*). These researchers observed that spirochetes were commonly present in the midgut of infected ticks and occasionally seen in the hindgut and rectal ampule. No spirochetes were observed in other tissues, including the salivary glands. During this initial period of study of Lyme disease spirochetes, how vector ticks actually transmitted this new pathogen was the subject of much discussion. Because spirochetes were reported to be restricted to the digestive tract of ticks, some investigators speculated that transmission occurred by tick regurgitation or fecal contamination (*6*). These inefficient routes of transmission became a less likely explanation after spirochetes were described in the hemolymph (*7*) and salivary glands of feeding ticks (*8*). The hypothesis that Lyme disease spirochetes were transmitted via the salivary gland route was confirmed when spirochetes were actually identified in tick saliva (*9*).

### Spirochete Multiplication

The principal tick vectors of Lyme disease spirochetes in North America are *I. scapularis* and *I. pacificus*; the developmental stage of the former species that transmits most human infections is the nymph. Although transmission by adult *I. scapularis* or transovarially infected larvae remains possible, our review focuses on tick-spirochete interactions within nymphal *I. scapularis*. Larval ticks ingest spirochetes from infected reservoir hosts, molt, and emerge as nymphs. When spirochetes are ingested by larvae, they rapidly multiply in the replete tick until the nymphal molt, when a precipitous drop in spirochete numbers occurs (*10*,*11*). Thus, at the time questing nymphs are likely to contact their potential victims, spirochete numbers are at their lowest and generally restricted to the lumen of the midgut. When nymphal feeding begins, a pronounced multiplication of spirochetes takes place in the tick. Nymphal *I. scapularis* take approximately 3 to 4 days to complete feeding. Spirochete numbers are reported to increase >300- fold during this feeding period, increasing from a mean of 496 spirochetes in unfed nymphs to 166,575 at 72 hours after attachment (*12*). Along with this rapid multiplication, other changes are taking place in the spirochete population that may lay the groundwork for eventual transmission to the host.

### Spirochete Surface Proteins

Virtually all spirochetes in the midgut of an unfed nymph express outer surface protein (Osp) A. This protein is also the predominant surface antigen expressed by the spirochetes in vitro. As the nymphal ticks start to feed and the spirochetes in the midgut begin to multiply rapidly, most spirochetes cease expressing OspA on their surface (*13*,*14*). Simultaneous with the disappearance of OspA, the spirochete population in the midgut begins to express a different Osp, OspC (*15*). Upregulation of OspC begins during the first day of feeding and peaks 48 hours after attachment (*14*). After this time point, the proportion of spirochetes in the midgut expressing OspC decreases rapidly. Therefore, the repression of OspA synthesis and upregulation of OspC correlate with the exit of spirochetes from the midgut, dissemination through the hemolymph, and passage through the salivary glands of the feeding tick. Several researchers have hypothesized that OspA binds spirochetes to the tick midgut (*14*,*16*,*17*). By downregulating OspA, the spirochetes might free themselves to migrate through the midgut epithelium and out of the midgut. Recently, purified OspA of *B. burgdorferi* was shown to bind to suspensions of tick midgut cells. The binding domains apparently reside in both the central and carboxy-terminus of the OspA protein; OspA also binds to itself (*16*). Thus, a picture emerges of aggregates of OspA-positive spirochetes bound to the tick midgut. Also, *B. burgdorferi* and *B. afzelii* expressing OspA were shown recently to adhere to tick-cell cultures more readily than spirochetes not producing this protein (*17*). Thus, when OspA is rapidly cleared from the spirochete surface, a proportion of the spirochete population may be free to leave the midgut and migrate to the salivary glands for transmission in saliva. The tick midgut protein that binds to OspA has not yet been described but should be an area of future research, as will be identifying factors that modulate the dispersal of spirochetes from the tick midgut (*18*).

Factors that regulate the switch from expression of OspA to OspC are likely varied and complex. Temperature is clearly one factor. As the tick finds a host and starts to feed, it moves from ambient temperature to the temperature at the surface of mammalian host skin. This rapid change in temperature clearly influences the spirochete population. Shifting spirochetes in vitro from lower temperatures to 37°C induced OspC expression (*14*,*19*). Similarly, other spirochete proteins, such as the Erp lipoprotein family, are upregulated during temperature increase (*20*). Cell density also may regulate spirochete protein expression. In a series of experiments using anti-OspA antibody passively transferred to mice serving as hosts for infected nymphal ticks, decreased spirochete density resulting from antibody-mediated death was associated with a lack of expression of OspC (*21*). Similarly, growth phase affects the synthesis of various proteins, including OspC (*22*). A change in pH in vitro can influence the expression of many proteins, including OspC (*23*,*24*). When ticks ingest blood, the pH of the midgut decreases from 7.4 to 6.8, which acts interdependently with increasing cell density and increased temperature to promote the reciprocal expression of OspA and OspC (*25*). The expression locus of the variable-like sequence (*vlsE*) may also prove to be of interest, since heterogeneity at this site appears to increase when ticks start to feed (*26*).

### Dissemination to the Salivary Glands

Different experimental strategies have been used to visualize spirochetes as they move from the tick midgut, through the hemolymph, and into the salivary glands before delivery to the host. Spirochetes present in hemolymph (*7*) and salivary glands have been directly visualized by electron microscopy (*8*). Indirect methods for detecting spirochetes in tick salivary glands included salivary gland explant cultures in BSK medium (Sigma Chemical Co., St. Louis, MO) and an infectivity assay of salivary gland homogenates inoculated into mice (*27*). These experiments demonstrated that although spirochetes were occasionally detected in salivary glands early during tick feeding, sufficient numbers of infectious spirochetes were not present within the salivary glands to cause infection in experimental hosts until at least 60 hours after tick attachment. Recently, confocal microscopy has allowed direct determination of specific spirochetal proteins synthesized in salivary glands. This approach has shown that small populations of spirochetes expressing OspA are present in the tick salivary glands and the dermis at the site of tick feeding early during the tick bloodmeal, but these spirochetes do not appear to be infectious. After 2 days of feeding, large numbers of spirochetes belonging to two predominant populations appear in the salivary glands and local dermis of the feeding site: spirochetes expressing only OspC, and spirochetes expressing neither OspA nor OspC (*26*). Infection of the host may be determined by actual numbers of spirochetes, the particular Osp phenotype entering the host from the tick, or both.

### Transmission to Host and Duration of Tick Feeding

Most persons bitten by nymphal *I. scapularis* do not become infected with *B. burgdorferi* and do not become ill with Lyme disease. Although 25% to 30% of nymphal *I. scapularis* in the northeastern United States are infected with *B. burgdorferi* sensu stricto, only approximately 1% to 2% of persons with recognized bites by nymphal *I. scapularis* become infected. One reason for this low rate of infection is that most ticks are detected and removed before they transmit infectious spirochetes. Virtually no transmission occurs during the first day of nymphal feeding, inefficient transmission takes place during the second day of tick feeding, and transmission is extremely efficient during the third day of nymphal feeding (*28*,*29*). These observations are consistent with the timing of spirochete multiplication, switching of Osps, and dispersal within the tick. A basic understanding of tick-spirochete interactions and transmission dynamics clearly has important implications in the clinical setting. The fact that prompt tick removal aborts transmission of *B. burgdorferi* sensu stricto is an important reason why most physicians in Lyme disease-endemic areas do not prophylactically treat persons bitten by ticks. In Europe and Asia, where other tick species (*I. ricinus* and *I. persulcatus*) transmit several genospecies of spirochetes, the situation may be more complex, with some risk of transmission during the first day of tick attachment (*30*,*31*).

###  Vaccine Implications

An important practical outgrowth of understanding how *B. burgdorferi* populations change during tick feeding is the enhanced insight into the molecular mechanisms of how the human Lyme disease vaccine based on OspA (*32*,*33*) works. Early on it was noticed that when anti-OspA antibody was present in the host at the time of tick attachment or during the first 24 hours of tick feeding, infection was prevented. If, however, antibodies were passively transferred to the host after this window of opportunity, infection was not prevented or cured with anti-OspA antibody (*13*,*34*). The implication was clear: spirochetes were killed inside the tick, before transmission to the host. Originally, it was suggested that anti-OspA antibodies actually sterilized the tick, eliminating all spirochetes present before they were transmitted (*13*). Subsequent studies with variable levels of anti-OspA antibody showed that ticks were only occasionally sterilized, at the highest levels of antibody in an immune host. More subtle effects were demonstrated with passively transferred anti-OspA antibody, which demonstrated that spirochete populations in the midgut were diminished but not eliminated. This diminution also prevented the switch from OspA expression to OspC expression and prevented dispersal of spirochetes to the salivary glands and transmission to the host (*21*). Thus, the action of anti-OspA antibody ingested by the tick had subtle effects on spirochete populations that blocked transmission to the host. Anti-OspC immunity appears to differ, acting in both the invertebrate and vertebrate host (*34*,*35*). These studies are a prime example of how insight into basic vector-pathogen interactions can lead to development of important prevention tools used to combat human disease.

### Tick-Borne Relapsing Fever

In 1857, Livingstone published his observations resulting from 16 years of exploration in southern Africa. In regions that are now Angola and Mozambique, he documented an illness of humans following the bite of a tick, known regionally as the “tampan” or “carapato.” This brief description was the first written account of tick-borne relapsing fever caused by *B. duttonii*. The tick was described by Murray in 1877 and named *Argas moubata*, later revised within the genus *Ornithodoros*. In 1905, Dutton and Todd (*36*) reported that *O. moubata* transmitted spirochetes to monkeys while feeding on them--the first demonstration that ticks were capable vectors of relapsing fever spirochetes. Also included in this landmark work was the observation that spirochetes were present in both the midgut and malpighian tubules of infected ticks.

Relatively few studies have examined the distribution of relapsing fever spirochetes in tissues of argasid ticks. Most early investigations examined *B. duttonii* in *O. moubata,* expanding on the work of Dutton and Todd, and demonstrated spirochetes in numerous tick tissues including the midgut, synganglion (central ganglion), malphigian tubules, salivary glands, ovaries, and coxal organs. Early investigators also demonstrated that *B. duttonii* is transmitted by contamination of infected coxal fluid and tick bite. Burgdorfer’s study of *B. duttonii* in nymphal and adult *O. moubata* is one of the most thorough investigations of any relapsing fever spirochete (*37*). While confirming the infection of many tick tissues, Burgdorfer also showed that *B. duttonii* enters the hemolymph as early as 24 hours after ticks acquire spirochetes in their midgut by feeding on a spirochetemic mouse. Also, the mode of transmission varies with the stage of tick. Nymphal *O. moubata* transmit *B. duttonii* in the saliva; adults transmit primarily via the coxal fluid. This stage-dependent difference in the primary mode of transmission helped clarify earlier observations.

### *B. hermsii*-Tick Interactions

Tick-borne relapsing fever was first reported in the United States in 1915, following the recognition of five human patients in Colorado (*38*). The tick vector for the causative spirochete lives in the higher elevations in western North America and was named *O. hermsi* in 1935 (*39*). Relapsing fever spirochetes transmitted by *O. hermsi* were first named *Spirochaeta hermsi* in 1942 and later changed to *Borrelia hermsii* in 1948. The criterion for naming the spirochete *B. hermsii* was based on the many observations in the laboratory by Davis (*40*). *O. hermsi* was capable of transmitting this spirochete while other species of ticks, *O. turicata* and *O. parkeri*, were not, although these other species of ticks were capable of transmitting other species of spirochetes (*40*). The mechanisms responsible for this strict species specificity for the transmissibility of one species of spirochete by only one species of tick are unknown.

*B. hermsii* infects a variety of small mammals living in coniferous forests at moderate to high elevations. The primary hosts for spirochetes and ticks are diurnal rodents such as chipmunks and tree squirrels. Ticks living in the nests or crevices nearby feed on these hosts at night, taking their bloodmeal quickly within 15 to 90 minutes, then retreating to their off-host refuge. These ticks and other species of *Ornithodoros* can fast for months to many years and retain infectious spirochetes throughout a life cycle that may take years to complete (*41*). Therefore, ticks have a greater potential to maintain spirochetes in nature for prolonged periods than do individual mammalian hosts that are infective to new cohorts of ticks while intermittently spirochetemic for 14 to 30 days (*42*).

Although other, larger species of *Ornithodoros* excrete substantial amounts of coxal fluid while feeding, *O. hermsi* excretes little or none while on the host. Therefore the only efficient mechanism for *O. hermsi* to transmit *B. hermsii* is by bite (*43*). The larva, three to five nymphal stages, and adult *O. hermsi* are all capable of transmitting *B. hermsii* (*43*,*44*). Transovarial transmission is rare (*43*), so larvae in nature are unlikely to be infective during their blood meal.

The distribution of *B. hermsii* in organs and tissues of *O. hermsi* has received little attention. In 1942, Wheeler published the only study before 1998 that examined different tissues of *O. hermsi* for *B. hermsii* (*45*). Spirochetes were consistently found in the midgut but only occasionally seen in the hemocoel, muscles, and “esophagus” for up to 38 days after infection was initiated in ticks. No spirochetes were seen in the salivary glands. Yet, based on recent observations (*46*), Wheeler’s negative data were most likely due to his use of thin sections and silver stain. The lack of specific immunologic stains for fluorescence microscopy 60 years ago made visualizing spirochetes much more difficult than today. Schwan and Hinnebusch (*46*) examined 41 *O. hermsi* from 33 to 144 days after infection with *B. hermsii* and found that the salivary glands from all ticks were infected. The midgut from 33 ticks and the synganglion from 22 ticks were also examined, and spirochetes were present in these organs from all ticks.

### Antigenic Variation in Mammals and Ticks

**Figure 2:**
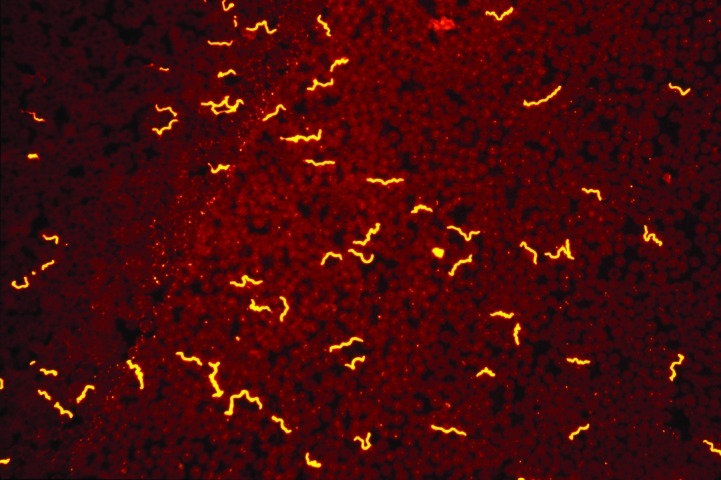
*Borrelia hermsii* visualized with an immunofluorescent stain in a thin blood smear of an experimentally infected mouse. Such recurrent, high densities of spirochetes circulating in the peripheral blood of small mammals allows for the acquisition of these bacteria by fast-feeding ticks that ingest a small volume of blood.

During the last 20 years, many studies have examined the mechanism of antigenic variation of *B. hermsii,* which has been proclaimed as a spirochete adaptation to evade the mammalian host’s immune response (*47*). The ability of the spirochete to evade immunologic destruction through antigenic variation within the mammal allows for the prolonged, recurrent bacteremias (Figure 2) that make spirochetes accessible to fast-feeding ticks and hence facilitate horizontal transmission of spirochetes in nature. A single cell of *B. hermsii* can give rise to 30 antigenic variants, each of which expresses a unique, variable major protein (Vmp) that confers a specific serotype (*47*,*48*). Other than the suggested role of immune evasion, no function has been demonstrated for these Vmps, which occur in two size classes, the variable large proteins (Vlps) and the variable small proteins (Vsps) (*49*). During mammalian infection, *B. hermsii* produces cyclic spirochetemias that may achieve a density of 10^8^ bacteria/ mL of blood. Each acute phase of spirochetemia contains a population of spirochetes composed almost entirely of one serotype (*48*). Relapse populations are predominated by a serotype different from the population preceding or following it.

**Figure 3:**
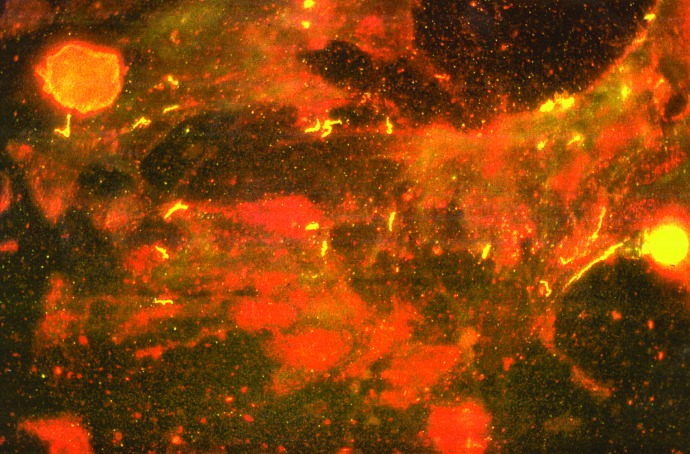
*Borrelia hermsii* visualized with an anti-variable small protein (Vsp) 33 antibody in a squashed salivary gland of *Ornithodoros hermsi*. Persistent infection of the salivary glands allows these spirochetes to be transmitted in only minutes while these ticks feed.

To address the influence of tick infection on the antigenic stability of spirochetes, two cohorts of *O. hermsi* were each infected with a different serotype of *B. hermsii*, serotype 7 or serotype 8 (*46*). The ticks were allowed to molt to the next stage and then fed individually on single mice. Eighteen (19%) of the 95 ticks transmitted spirochetes, and with every infection the first spirochetemia in mice had the same serotype ingested previously by the tick. Additional groups of ticks infected with the same serotypes were also examined. Polymerase chain reaction (PCR) and restriction fragment length polymorphism analyses of the expression locus for the *vmp* genes showed that either the *vlp7* or *vsp8* gene was present, corresponding to the serotype of *B. hermsii* ingested by the tick. Cumulatively, these observations suggested that no serotype-related antigenic changes occurred in the spirochetes during infection in ticks. However, when single salivary glands from infected ticks were examined by immunofluorescence microscopy with anti-Vlp7 or anti-Vsp8 antibody, no spirochetes were seen. Immunofluorescence staining of the other salivary glands with other antibodies showed, however, that all the glands were infected with spirochetes and that the bacteria were now expressing Vsp33, an Osp known previously from only one culture-adapted strain of *B. hermsii* (*50*). When blood from a mouse infected with serotype 8 of *B. hermsii* was inoculated into culture medium and incubated at either 23°C or 37°C, spirochetes continued to express Vsp8 at 37°C but switched to Vsp33 at 23°C (*46*). Thus, both a transfer of spirochetes from the mammalian bloodstream to tick and a drop in environmental temperature in vitro brought about an antigenic switch from bloodstream Vmp to Vsp33. Recently, double-label immunofluorescence staining of spirochetes in the midgut and salivary glands of *O. hermsi* demonstrated that spirochetes expressing Vsp33 were detectable ≥28 days after ticks became infected (Figure 3). By 68 days after infection, essentially all the spirochetes in the salivary glands expressed Vsp33, while only a third of the spirochetes in the midgut did so.

### Species Comparisons

From the observations reviewed above, one phenomenon shared by *B. burgdorferi* and *B. hermsii* is the synthesis of OspC and Vsp33 at the time these spirochetes are transmitted by tick bite. DNA and amino acid sequence analysis also shows that these proteins are homologous (*51*) and that antisera produced to the two proteins are cross-reactive (*51*–*53*). Several other species of *Borrelia* also contain a related gene or protein recognized with either Northern or Western blot (*53*). Therefore, this family of surface proteins may be shared by all species of borreliae, which are spirochetes defined, in part, by their requirement for an arthropod vector for transmission. Our hypothesis is that these proteins are involved in the transmission of these spirochetes from tick to vertebrate host.

**Figure 4:**
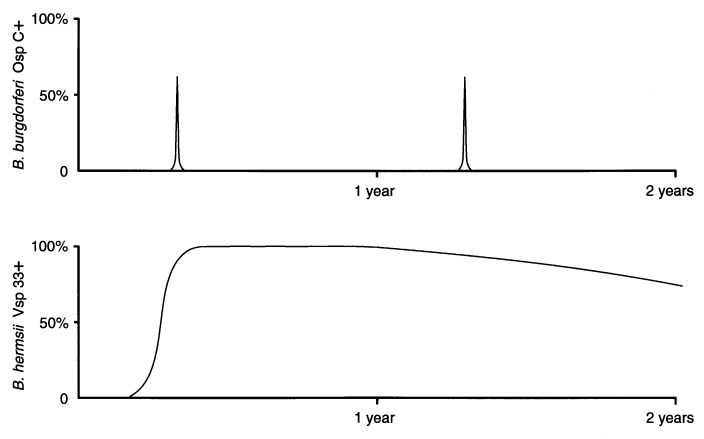
Schematic representation of the presence of OspC on *Borrelia burgdorferi* and variable small protein (Vsp) 33 on *B. hermsii* during infection in their respective tick vectors. Shown is the proportion of spirochetes detected with double-label immunofluorescence stains that includes either anti-outer surface protein C (Osp C) antibody (top) or anti-Vsp33 antibody (bottom). *B. burgdorferi* produces OspC in the midgut of *Ixodes scapularis* for only a few days, starting after these ticks have attached and begun to feed. During the 3 to 5 days of tick feeding, these spirochetes replicate, disseminate from the midgut, and are transmitted via saliva. In contrast, *B. hermsii* gradually upregulates the synthesis of Vsp33 after infecting *Ornithodoros hermsi*, and essentially all the spirochetes express the protein during persistent infection of the tick salivary glands.

The temporal synthesis of OspC and Vsp33 during spirochete infection in ticks is strikingly different between *B. burgdorferi* and *B. hermsii,* but appears to be adaptive to ixodid versus argasid ticks, which have considerably different feeding behaviors. Most nymphal *I. scapularis* take 3 to 4 days to feed whereas *O. hermsi* feed to repletion in only 15 to 90 minutes. In free-living, unfed *I. scapularis*, *B. burgdorferi* is usually found only in the midgut and OspC is not expressed. However, following tick attachment *B. burgdorferi* replicates, downregulates OspA, disseminates from the midgut to salivary glands, synthesizes OspC, and is transmitted via the saliva after 2 to 4 days of feeding. An increase in temperature and the ingestion of blood, environmental cues associated with a free-living tick having attached and begun to feed on a host, stimulate a subpopulation of *B. burgdorferi* to transiently synthesize OspC during feeding. If OspC is required for transmission of *B. burgdorferi* by tick bite, there is ample time, because of the slow feeding behavior of *Ixodes* ticks, for both the dissemination of spirochetes from the midgut to the salivary glands and the novel synthesis of OspC. Numerous studies have shown that humans and experimental animals infected by tick bite seroconvert to OspC early, demonstrating that this protein is expressed in mammals for some undetermined period. In free-living, unfed *O. hermsii*, the distribution of *B. hermsii* in these ticks and their expression of Vsp33 are just the opposite, with spirochetes established in the salivary glands and nearly all expressing Vsp33. In the scenario with *O. hermsi* feeding for only minutes after encountering a host, there is no time for spirochetes to disseminate out of the midgut, penetrate the salivary glands to the salivary duct, and also synthesize a new Osp that may facilitate (or be required for) transmission. Hence *B. hermsii* is in a constant state of readiness for transmission that is comparable to the phenotype and localization displayed by *B. burgdorferi* only briefly during a few days of attachment by *I. scapularis* (Figure 4).

The phenotypic changes and dissemination shown by *B. burgdorferi* during transmission by tick bite point to several possible functions for OspC: dissemination from the midgut, infection of the salivary glands, or successful colonization in the mammal following delivery into the feeding lesion in the dermis. Because the changes in protein synthesis and movement of the spirochetes occur rapidly in the nymphal ticks, identifying the precise time when OspC is produced in relation to the spirochetes’ movement in the tick is difficult to determine by microscopy. Quantitative reverse transcription-PCR may help increase sensitivity through detecting specific gene transcripts in different tick tissues sampled a different times. However, the events shown by *B. hermsii* during its dissemination in the tick are much more protracted and may shed some light on the function of OspC, Vsp33, or other proteins. Because it takes approximately 3 weeks or more for *B. hermsii* to disseminate and become established in the salivary glands of *O. hermsi*, immunofluorescent staining of these spirochetes in tick tissues at successive intervals after infection has recently shown that *B. hermsii* can infect the salivary glands before the upregulation of Vsp33. At least for this relapsing fever spirochete, Vsp33 does not appear to be needed either for dissemination from the gut or invasion of the salivary glands.

Additional species of *Borrelia* and their expression of Osps associated with transmission need to be examined. If the OspC, Vsp33, or other proteins are required for transmission by ticks, we anticipate finding homologs to these proteins in other species associated with their transmission. Other relapsing fever spirochetes in *Ornithodoros* ticks, *B. anserina* in *Argas* ticks, and other borrelia associated with ixodid ticks will be fruitful tick-spirochete associations to study. A comparison of borrelial genomes will also be helpful when such sequences become available. Finally, with important advances being made recently to inactivate and introduce genes in these spirochetes (*54*,*55*), experiments to examine the importance of specific proteins in various steps of the transmission cycle are on the horizon.
